# Discordance for genotypic sex in phenotypic female Atlantic salmon (*Salmo salar*) is related to a reduced *sdY* copy number

**DOI:** 10.1038/s41598-020-66406-x

**Published:** 2020-06-15

**Authors:** Morgan S. Brown, Brad S. Evans, Luis O. B. Afonso

**Affiliations:** 1 School of Life and Environmental Sciences, Centre for Integrative Ecology, Deakin University Warrnambool Campus, Warrnambool, Victoria 3280 Australia; 2 Breeding & Research, Tassal Operations, Hobart, Tasmania 7000 Australia

**Keywords:** Genetics, Molecular biology

## Abstract

The master sex determinant in rainbow trout (Oncorhynchus mykiss), sexually dimorphic on the Y chromosome (*sdY*), is strongly but not perfectly associated with male phenotype in several other species from the family Salmonidae. Currently, the cause and implications of discordance for *sdY*-predicted genotypic sex and phenotypic sex in these species is unclear. Using an established multiplex PCR test for exons 2 and 3 of *sdY*, we demonstrated that *sdY*-predicted genotypic sex was discordant with histologically evidenced phenotypic sex in 4% of 176 Tasmanian Atlantic salmon. All discordant individuals were phenotypic females presenting a male genotype. Using real-time qPCR assays that we developed and validated for exons 2, 3 and 4 of *sdY*, all genotype-phenotype discordant females were confirmed to possess *sdY*, albeit at a reduced number of copies when compared to phenotypic males. The real-time qPCR assays also demonstrated reduced levels of *sdY* in 30% of phenotypic females that the established multiplex PCR-based test indicated to be devoid of *sdY*. These findings suggest *sdY* may be reduced in copy number or mosaicked in the genomic DNA of *sdY*-positive phenotypic female Atlantic salmon and highlight the importance of understanding the effects of reduced *sdY* copies on the development of phenotypic sex.

## Introduction

Many members of the family Salmonidae (comprised of three sub-families: Coregoninae, Thymalline and Salmoninae) are of high societal and economic importance for their value in aquaculture, wild stock fisheries and recreational sports fisheries^1,2^. With particular reference to Atlantic salmon (*Salmo salar*) and rainbow trout (*Oncorhynchus mykiss*), they also present as important scientific models in the fields of toxicology, immunology, physiology, nutrition and genetics^3–5^.

The development of a reliable DNA-based method for identifying genotypic sex in salmonids has been of high interest to facilitate management of native populations and production of farmed stocks^6–9^. Initial efforts resulted in the discovery of several markers tightly linked to the sex determining locus on the Y chromosome within the *Oncorhynchus* genera of the Salmonidae. These include OtY1 in chinook salmon (*Oncorhynchus tshawytscha*)^10^, GH-Y in chinook^11^, coho (*Oncorhynchus kisutch*), masu (*Oncorhynchus masou*)^12^, chum (*Oncorhynchus keta*) and pink salmon (*Oncorhynchus gorbuscha*)^13^, and OmyY1 in rainbow trout^14^. However, variable rates of genotype-phenotype discordance using these sex markers have been reported^14–20^. This is likely attributed to the fact that these markers are non-functional sequences residing close to the sex determination locus, and thus are not directly selected for^20^.

Recently the master sex determining gene sexually dimorphic on the Y chromosome (*sdY*) was discovered in rainbow trout, and its presence in genomic DNA (gDNA) was demonstrated to align 100% with male phenotype in this species (425 individuals studied)^21^. *sdY* is also strongly associated with male phenotype in several other species from all three subfamilies of the family Salmonidae, suggesting a conserved function for this gene as the master sex determinant^9^. However, cases where *sdY* is not perfectly associated with male phenotype have been reported in populations of lake char (*Salvelinus namaycush*), chinook salmon^9,22^, sockeye salmon (*Oncorhynchus nerka*)^23^ and Atlantic salmon^24–27^. In addition, in European whitefish (*Coregonus lavaretus*) and lake whitefish (*Coregonus clupeaformis*), both phenotypic males and females possess *sdY*^9^. The cause and significance for these cases of *sdY*-predicted genotype-phenotype discordance remain unclear, however some studies have suggested phenotype miscalls, sex reversal and/or loss of gene function in select individuals could be attributed^22,27^. For European whitefish and lake whitefish (members of the Coregoninae), *sdY* may act through a dosage mechanism to trigger testicular differentiation^9^. Indeed, variations in sequence copy number have been reported for multiple non-functional markers on the Y chromosome in salmonids^20^. Thus, quantitative approaches may provide new insights in the association of *sdY* with phenotypic sex in salmonids.

Studies in the Tasmanian Atlantic salmon population (originated from the River Philip in Nova Scotia, Canada) have consistently identified individuals whose phenotypic sex does not concur with *sdY* predictions^24,27^. This makes the Tasmanian population ideal for studying the association of *sdY* copy number with phenotypic sex. In the present study, we determined the rate of concordance for *sdY*-predicted genotypic and phenotypic sex in 176 Tasmanian Atlantic salmon using a multiplex PCR-based test adapted from Eisbrenner, *et al*.^27^. This method for determining genotypic sex in Atlantic salmon has also been used for studies in sexual maturation^28–31^, sex differentiation^32^, farmed stock introgression^33–35^ and fisheries management^36^. Phenotypic sex for all fish was determined by histology. We then examined the abundance of *sdY* in gDNA for all fish using real-time quantitative PCR (qPCR) assays for exons 2, 3 and 4 of *sdY*. Our findings confirm that a proportion of phenotypic females possess *sdY* in the Tasmanian Atlantic salmon population. However, *sdY* appears to be reduced in copy number in these individuals compared to phenotypic males. No phenotypic males lacking *sdY* were observed in this study.

## Results

### Histological assessment of phenotypic sex

**Figure 1:**
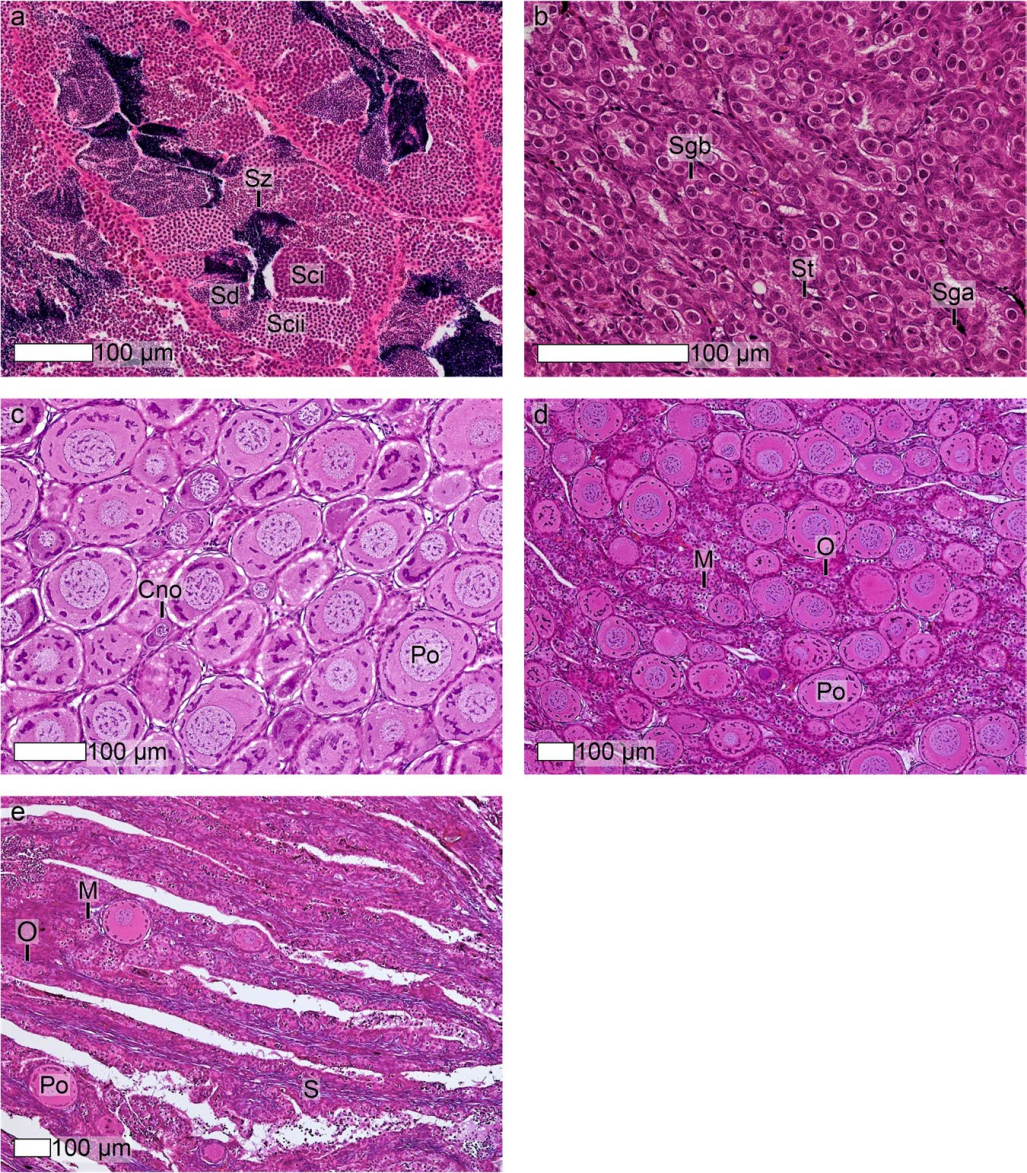
Histological assessment of phenotypic sex. Gonadal phenotypes observed amongst the 176 Tasmanian Atlantic salmon used in this study. (**a**) testis with cells at all spermatogenic stages; (**b**) testis in resting state, comprised of type A and type B spermatogonias, and sertoli cells; (**c**) ovary almost entirely comprised of perinucleolar oocytes; (**d**) ovary comprised equally by perinucleolar oocytes and cysts of oogonias and oocytes in meiotic prophase; (**e**) ovary dominated by stromal tissue and cysts of oogonias and oocytes in meiotic prophase. Sga, type A spermatogonia; Sgb, type B spermatogonia; Sci, primary spermatocytes; Scii, secondary spermatocytes; Sd, spermatids; Sz, spermatozoa; St, sertoli cell; O, oogonias; M, oocytes in meiotic prophase; Cno, chromatin nucleolar oocyte; Po, perinucleolar oocyte; S, stromal cells.

104 males and 72 females were identified in the population sampled. Two distinct male phenotypes were observed. The gonads of 33 males were comprised of seminiferous tubules predominantly filled with cysts of primary and secondary spermatocytes and spermatids. Spermatozoa typically filled the lumen of the seminiferous tubules, and sertoli cells and type A and B spermatogonias were adjacent to the seminiferous epithelium (Fig. 1a). Gonads from the remaining 71 males were characterised by cysts of type A and B spermatogonias and sertoli cells (Fig. 1b). For the majority of females sampled, the gonad was predominantly comprised of oocytes in the early perinucleolus stage, with chromatin nucleolar oocytes, oocytes in meiotic prophase and oogonias also present (Fig. 1c). However, in four females perinucleolar oocytes occupied a much smaller proportion of the gonad (0–50%), which was instead comprised mainly of cysts of oogonias and oocytes in meiotic prophase. Large amounts of stromal tissue were also identified in these gonads (Fig. 1d,e).

### Genotype-phenotype discordance identified using multiplex PCR-based test

Male genotype was demonstrated for all phenotypic males in the population studied. Female genotype was evident in 65 of the 72 phenotypic females studied, the remaining seven had a male genotype. Five of the 65 genotype-phenotype concordant females amplified PCR products for *fabp6b* and exon 3 of *sdY*, but not exon 2 (Table 1). The remaining 60 genotype-phenotype concordant females amplified PCR products for *fabp6b*, but not exon 2 or exon 3 of *sdY*. No other genotypes were observed. Of the seven phenotypic females with a male genotype, two had ovaries comprised mainly by cysts of primary oocytes in prophase of meiosis I and stromal tissue (Fig. 1d,e). All of the phenotypic females that amplified PCR products for *fabp6b* and exon 3 of *sdY*, but not exon 2, had ovaries dominated by perinucleolar oocytes.

**Table 1:** Multiplex PCR-based test for identifying genotypic sex of Atlantic salmon.

Genotype	Phenotype	
	Male	Female
Male (*fabp6b* & exon 2 + 3 *sdY* positive)	104	7
Female (*fabp6b* positive, exon 2 + 3 *sdY* negative)	0	60
Female (*fabp6b* & exon 3 *sdY* positive, exon 2 *sdY* negative)	0	5

### Reduced sdY copies detected in genotype-phenotype discordant individuals

**Figure 2:**
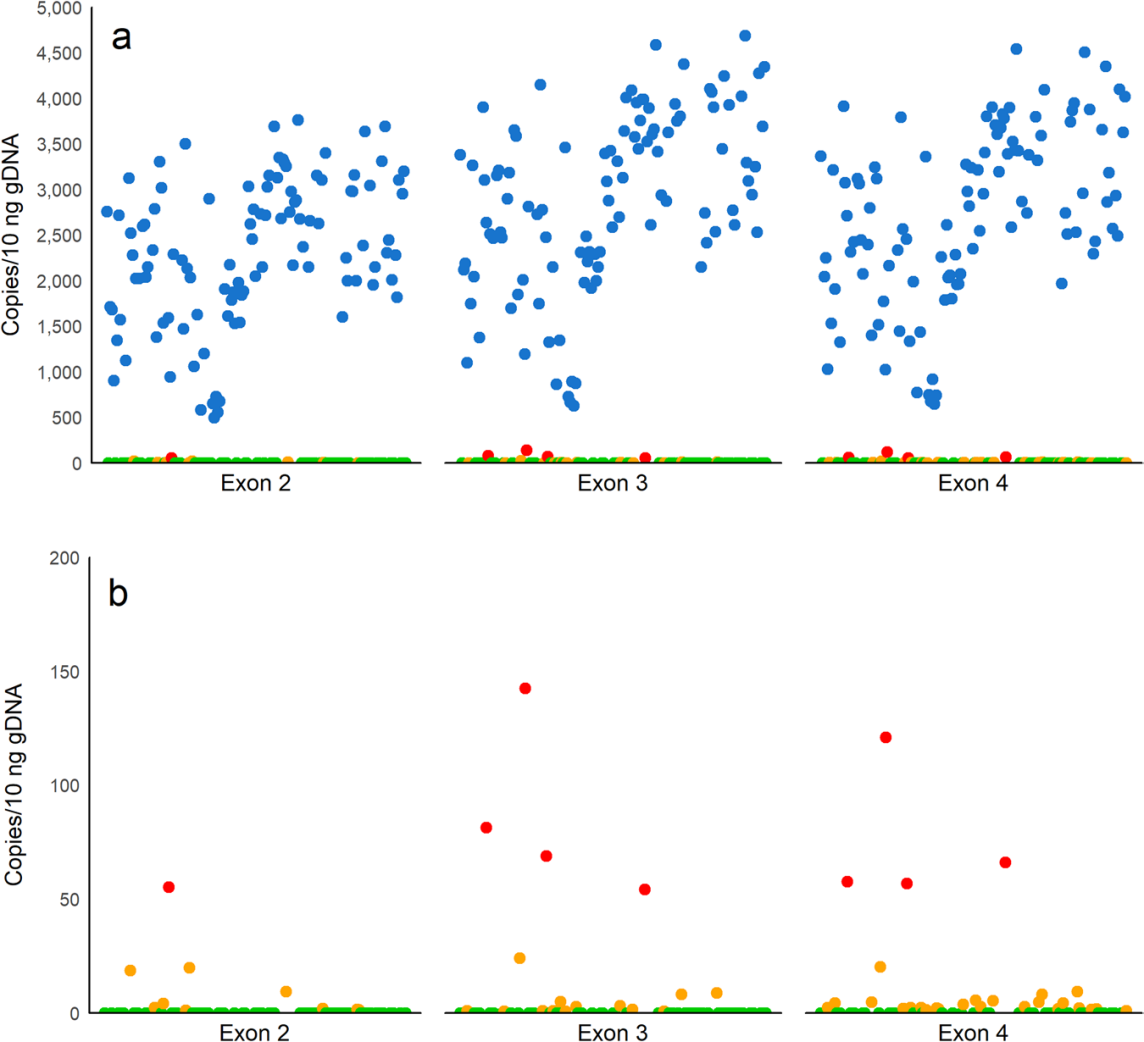
Quantification of *sdY* by real-time qPCR. Number of *sdY* copies detected within the linear dynamic range (LDR) of the real-time qPCR assay in phenotypic males () and phenotypic females (), and outside of the LDR in phenotypic females ().  indicates a phenotypic female with no *sdY* copies detected. (a) data for all 176 Tasmanian Atlantic salmon used in this study; (b) magnified view of individuals with low numbers of target copies.

**Figure 3:**
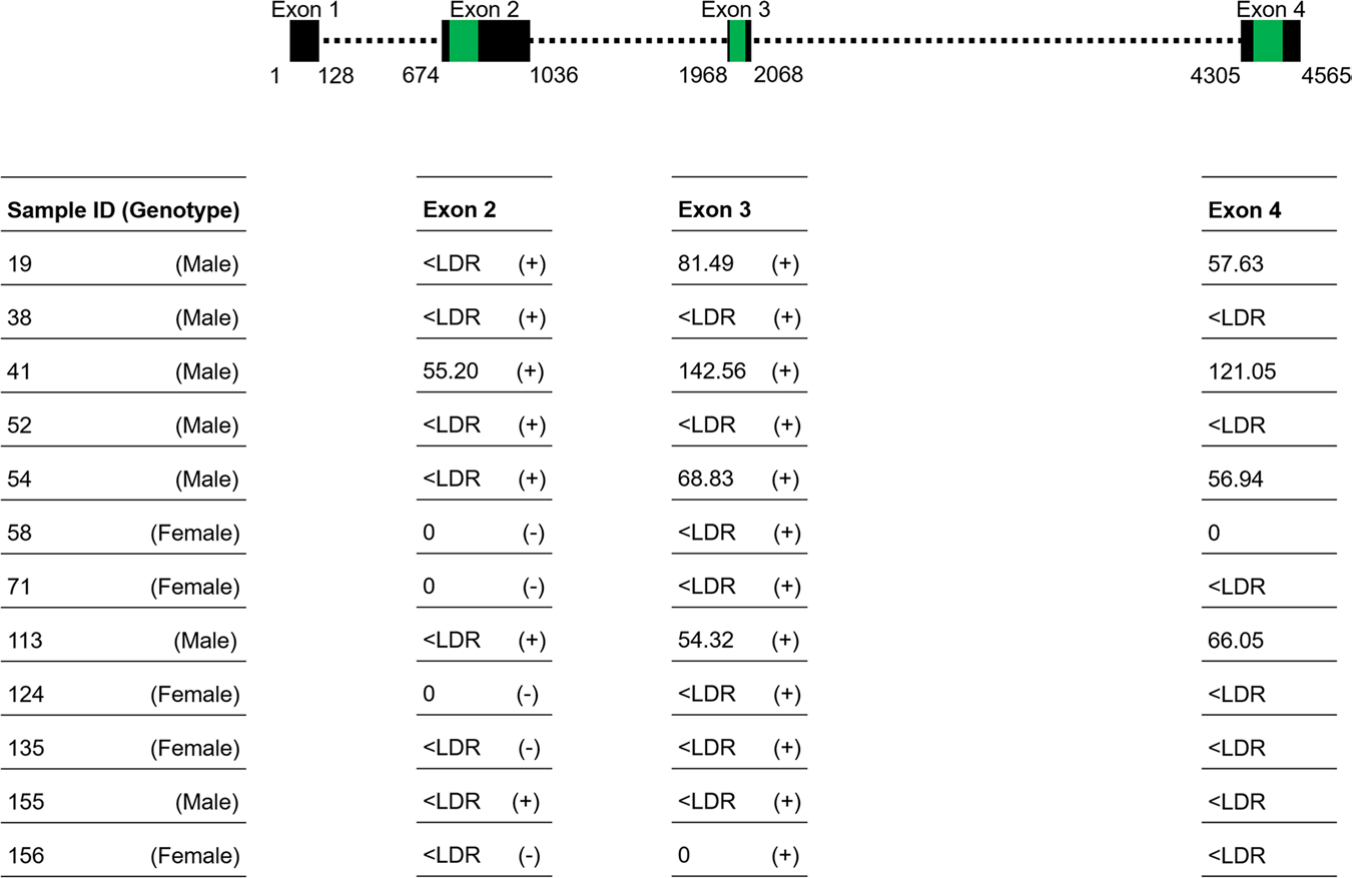
*sdY* gene structure, real-time qPCR assay amplicon localisation and results of the real-time qPCR assays for phenotypic females with an *sdY*-positive genotype (as determined by the multiplex PCR-based test). Structure of *sdY* in Atlantic salmon according to the gene sequence provided by http://www.ncbi.nlm.nih.gov (GenBank accession number: KT223110). The size and position of each exon (▪) and intron (▪▪▪) is indicated. The 5′ untranslated region (UTR) spans from positions 1–100, and the 3′UTR spans from positions 4395–4565. Amplicon locations for the real-time qPCR assays ( are indicated. Data presented as copies/10 ng DNA. < LDR, below the linear dynamic range of the real-time qPCR assay; (+), amplified in the multiplex PCR-based test; (−), did not amplify in the multiplex PCR-based test.

All gDNA samples from phenotypic males presented levels of exons 2, 3 and 4 of *sdY* within the linear dynamic range (LDR) of the real-time qPCR assays (Fig. 2a). A single phenotypic female sample had exon 2 in levels within the LDR, whereas four phenotypic females presented levels for exon 3 and exon 4 within the LDR. Exons 2, 3 and 4 of *sdY* were also detected in levels below the LDR in a considerable number of phenotypic females (Fig. 2b). The four phenotypic females presenting levels of exon 2, and/or exons 3 and 4 of *sdY* within the LDR were identified as four of the seven individuals for which genotype-phenotype was discordant. These exons were also detected in the remaining three individuals, but at levels below the LDR (Fig. 3). The mean number of copies/10 ng gDNA for exons 2, 3 and 4 of *sdY* in phenotypic males with a male genotype was 2,314.43 ± 789.13, 2880.99 ± 971.95 and 2725.83 ± 971.95, respectively. This was significantly higher than the mean of phenotypic females with a male genotype (excluding levels below the LDR), which was 55.20, 86.80 ± 33.59 and 75.42 ± 26.59 for exons 2, 3 and 4, respectively (exon 2: n = 104, t(103) = 29.20, p < 2.2e-16, one sample t-test; exon 3: n = 108, t(105.5) = −28.73, p < 2.2e-16; exon 4: n = 108, t(105.99) = −28.13, p < 2.2e-16, Welch’s t-test). The number of copies for exons 2, 3 and 4 of *sdY* were not significantly different between the two male phenotypes observed (exon 2: n = 104, t(102) = −0.26, p = 0.79, exon 3: n = 104, t(102) = −0.09, p = 0.93, exon 4: n = 104, t(102) = −0.07, p = 0.94; independent t-test).

**Figure 4:**
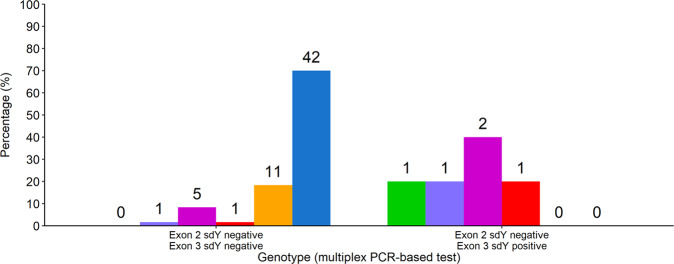
Combinations of *sdY* exons amplified in phenotypic females by real-time qPCR. Proportion of genotype-phenotype concordant females where exons 2, 3 and 4 (), 2 and 4 (), 3 and 4 (), 3 (), 4 () or no exons () of *sdY* were detected by real-time qPCR. Number of individuals observed is indicated above the respective bar.

In the five phenotypic females that had a female genotype characterised by absence of exon 2 but presence of exon 3 of *sdY* (as determined by the multiplex PCR-based test), at least one of the three exons investigated using real-time qPCR was present in gDNA at levels below the LDR (Fig. 3). Exon 2 was detected in two of these individuals by real-time qPCR despite not being detected by the multiplex PCR based test (Fig. 4). Low levels of predominantly exon 3 and/or exon 4 of *sdY* were also detected by real-time qPCR in several phenotypic females with a genotype devoid of *sdY* (as determined by the multiplex PCR-based test) (Fig. 4). None of the *sdY* exons were detected by real-time qPCR in the two genotype-phenotype concordant females whose ovaries were comprised mainly of cysts of oogonias and oocytes in meiotic prophase, and stromal tissue.

## Discussion

Using the multiplex PCR-based test, genotypic sex was concordant with phenotypic sex in 96% of the 176 fish studied. This rate of concordance is in agreement with findings from previous studies in the Tasmanian Atlantic salmon population, which used a larger sample size (up to 3,176 fish)^24,27^. In the present study, all genotype-phenotype discordant fish observed were phenotypic females with a male genotype. These findings contrast previous studies in the Tasmanian population, which have reported phenotypic males with a female genotype as the dominant form of genotype-phenotype discordance^27^. Therefore, the absence of phenotypic males with a female genotype in the present study is intriguing. *sdY* positive phenotypic females have been previously documented in Atlantic salmon from Tasmania^27^ and the Faroe Islands^26^, as well as in populations of north American sockeye salmon^23^ and chinook salmon^9,22^. In chinook salmon, similar findings to the present study were reported, whereby all phenotypic males investigated possessed the *sdY* gene as did a proportion (~2–22%) of phenotypic females. Alternatively, phenotypic males lacking *sdY* have been reported in north American sockeye salmon populations^23^, lake char^9^ and Norwegian Atlantic salmon^25^ in addition to the Tasmanian Atlantic salmon population^27^

Of the 65 genotype-phenotype concordant females observed in the present study, five amplified the product for exon 3 primers but did not for exon 2 using the multiplex PCR-based test. This phenomenon of an ‘incomplete *sdY* gene’ has been previously reported in Atlantic salmon from the Tasmanian population^27^, and is thought to be a result of instability in the 5’ end of *sdY* sequence^37^. Such an explanation may also provide insights in the occurrence of phenotypic female chinook salmon which possess exons 2, 3 and 4 of *sdY*, but not exon 1^22^. However, in the present study we found that some of the phenotypic females with a genotype characterised by an incomplete *sdY* gene in fact had very low intensity bands for exon 2 that were below the detection limit of Image Lab Software (Bio-Rad, USA). Considering these observations, we suspect that at least for some individuals the phenomenon of an incomplete *sdY* gene (as determined by the multiplex PCR-based test) can rather be attributed to a combination of low target amounts in template gDNA and the inclusion of ~7 times more primer for exon 3 than exon 2 in the multiplex PCR-based test used. In support of this, the band for exon 2 was much less intense than the band for exon 3 even when a male genotype was assigned. Reduced target amounts of the priming sites on exon 2 may suggest *sdY* is present at a reduced copy number in some phenotypic females in the Tasmanian Atlantic salmon population.

Variable copy numbers for *sdY* within salmonid populations have not previously been reported despite the use of real-time qPCR assays for detecting the presence of this gene in chinook salmon^22^ and Atlantic salmon^26^. However, variations in copy number for other Y- chromosome markers have been reported by several studies. In Chinook salmon, PCR-based tests for OtY1 and GH-Y have produced low intensity target bands in both phenotypic males and females^16,18^. Devlin, *et al*.^18^ also demonstrated reduced signal intensity for OtY8 (an 8 kb repeat containing the OtY1 sequence) by southern blot analysis in these individuals, further suggesting OtY1 was reduced in copy number. Alternatively, Muttray, *et al*.^20^ observed a single phenotypic male coho salmon and two phenotypic male pink salmon with increased signal intensity for GH-Y via southern blot analysis relative to other phenotypic males from the studied populations, suggesting GH-Y occurred in a higher copy number in these individuals. These results suggest the Y chromosome in salmonids is subject to multiple forms of sequence copy number at multiple loci^20^.

To test for variations in copy number of *sdY* within the Tasmanian Atlantic salmon population, we developed real-time qPCR assays for exons 2, 3 and 4 of *sdY*. An assay for exon 1 was not developed because this exon is considered to be comprised of unstable sequence^37^. The real-time qPCR assays confirmed the presence of *sdY* in all phenotypic males studied as well as the seven phenotypic females for which a male genotype was assigned by the multiplex PCR-based test. However, the number of target copies detected by each of the real-time qPCR assays was significantly less in phenotypic females with a male genotype compared to phenotypic males. The highest number of copies for each exon observed amongst phenotypic females was more than an order of magnitude lower than the mean number of copies detected in phenotypic males, and more than four times lower than the lowest number detected in a phenotypic male. These findings indicate *sdY* may occur at a reduced copy number in the genome of phenotypic female Atlantic salmon for which a male genotype has been assigned (using the multiplex PCR-based test) compared to phenotypic males.

Of the five phenotypic females that had a female genotype characterised by the presence of exon 3 but absence of exon 2 using the multiplex PCR-based test, two were demonstrated to in fact possess exon 2 using the real-time qPCR assay. In one of these five individuals, the real-time qPCR assays detected exons 2, 3 and 4. Alternatively, only exons 3 and 4 amplified in two other individuals, exons 2 and 4 in another, and only exon 3 in one other individual. However, the quantities of exons 2, 3 and 4 in template gDNA from these five individuals was below the LDR for the real-time qPCR assays. Failure to detect exon 2, exon 3, and exons 2 and 4, respectively, in four of these five individuals therefore most likely reflects the limitations of the real-time qPCR assays for reliably detecting very low quantities of target rather than these exons not being present in gDNA. Indeed, exon 3 was detected using the multiplex PCR-based test in the individual whom it was not detected using the real-time qPCR assay. Thus, results from the real-time qPCR assays confirm that the occurrence of individuals with a genotype characterised by an incomplete *sdY* gene is at least in some cases an artefact of the multiplex PCR-based test when low copies of target sequence are present in template gDNA. The real-time qPCR assays also detected the presence of *sdY* at levels below the LDR in 30% of genotype-phenotype concordant females that did not amplify any *sdY* products in the multiplex PCR-based test. Only a single exon of *sdY* was detected by real-time qPCR in the majority of these individuals (none amplified all three of the *sdY* exons tested). Also, a high proportion of the exons detected amplified only in a single well of the triplicate reactions.

Results from the real-time qPCR assays in the present study suggest *sdY* is present in a considerable proportion of phenotypic female Atlantic salmon from the Tasmanian population. To confirm these observations, DNA extractions and real-time qPCR runs were repeated for a number of samples from phenotypic females where target *sdY* sequence was detected by real-time qPCR. Repeated procedures were completed in the absence of samples from phenotypic males to mitigate *sdY* contamination. Comparable results were obtained for some but not all repeated samples (Supplementary Table S1), suggesting *sdY* cannot be consistently detected using the current methodology when present in a reduced quantity in gDNA. Devlin, *et al*.^18^ reported similar findings for the repeatability of detecting OtY1 and GH-Y at reduced levels in Chinook salmon. Thus, it’s possible that more phenotypic females from the present study in fact possessed *sdY* in a reduced number of copies but were not detected. Alternatively, all samples from phenotypic males (*sdY* present in high number of copies) showed a high level of repeatability when re-extracted and re-assayed (Supplementary Table S2). A reduced number of *sdY* copies and variable repeatability for the analysis in phenotypic females may suggest a ‘mosaic’ presence of *sdY* in the muscle cells of these individuals. In gDNA isolated from semen and sperm cells from infertile human males, a copy number less than one was detected for Sex-determining Region Y (*SRY*) using real-time qPCR, indicative of Y chromosome mosaicism^38^. Y chromosome mosaicism has also been demonstrated in blood cells from an aborted human foetus using fluorescence *in-situ* hybridisation^39^. In this study, some cells were completely devoid of signal for a Y chromosomal marker, whereas other cells showed varying signal intensities^39^. Considering ~3074 copies of the Atlantic salmon genome (GenBank accession number: GCA_000233375.4) are present in 10 ng gDNA, our findings suggest *sdY* is present in approximately a single copy in the male genome (mean copy number of 0.75, 0.94 and 0.89 determined for exons 2, 3 and 4, respectively, in phenotypic males with a male genotype). The *sdY* copy number estimate in phenotypic females with a male genotype is less than 0.1 (mean copy number of 0.02, 0.03, and 0.02 for exons 2, 3 and 4, respectively, excluding levels below the LDR). These estimates are in agreement with a previous study that reported male Atlantic salmon possess a single copy of the chromosomal region containing *sdY*^24^, and indicate that *sdY* is present in a single copy within this chromosomal region. These estimates also further suggest a mosaic presence of *sdY* in the muscle cells of some phenotypic females. Such a phenomenon would also explain the variability in amounts of *sdY* recovered from repeated gDNA extractions on the same sample. Understanding the mechanisms by which *sdY* mosaicism is acquired, and its effects on the development of phenotypic sex, both in parent and offspring, will be important to further our understanding of the association of *sdY* with phenotypic sex in Atlantic salmon.

The complete concordance of high number of *sdY* copies and male phenotype observed in the present study suggests *sdY* may be required above a threshold amount in gDNA to promote testicular development. A dosage-dependent mechanism for *sdY* has also been suggested in members of the Coregoninae family, following the observation that all phenotypic females and males possessed *sdY*, as determined by a PCR-based test^9^. However, the occurrence of phenotypic males with a female genotype described previously in Tasmanian^27^ and Norwegian Atlantic salmon populations^25^ suggests there could be alternative factors influencing sex determination in addition to *sdY* in this species. In support of this, we confirmed the occurrence of phenotypic males which lack *sdY* in the Tasmanian population using the real-time qPCR assays developed in this study and archived fin samples from known *sdY*-negative phenotypic male Tasmanian Atlantic salmon (Supplementary Table S3). These findings demonstrate that *sdY* quantification in gDNA does not explain genotypic discordance in phenotypic males, thus preventing the sole use of *sdY*-based quantitative methods to reliably indicate genotypic sex in Atlantic salmon. Future studies investigating the association of genotypic and phenotypic sex in salmonids should continue to assign phenotypic sex with histological evidence where possible. Additionally, observations of phenotypic sex at developmental stages prior to sexual maturity, including during the sex differentiation period, may provide new insights. For instance, genotype-phenotype discordance due to sex reversal could be evident during these stages.

## Methods

### Sampling

All procedures conducted during this trial were in accordance with approval by the Deakin University Animal Ethics Committee (Permit No. B29-2017), and were compliant with the guidelines outlined in the Australian code for the care and use of animals for scientific purposes (2013). This study used 176 (39.72 g ± 1.03) mixed-sex, freshwater acclimated juvenile Atlantic salmon from Salmon Enterprises of Tasmania Pty. Ltd. (SALTAS), Wayatinah, Tasmania. All fish were randomly selected for use in this study from a population that was being maintained under commercial conditions. Sampling was conducted over three consecutive days. Each fish was euthanized by a lethal dose of AQUI-S (AQUI-S, New Zealand). Once euthanized, mass was recorded for each fish followed by collection of dorsal muscle tissue and a single gonad. Muscle tissue was immediately placed in 500 µl of RNA*later* (Merck, Germany) and stored at ambient temperature for 5–7 days, then −80 °C for 12 months until time of analysis. The gonad was fixed in Bouin’s solution (Merck, Germany) for 5–7 days, then stored in 70% ethanol until time of processing.

### Histology

Fixed gonads were dehydrated by sequential immersion in 70%, 90%, and 100% ethanol, cleared with Xylene, embedded in Histosec wax (Merck, Germany) and sectioned with a microtome. For each sample, three 4 µm cross-sections were stained with hematoxylin and eosin then examined under a light microscope (10-400×). Paraffin processing, embedding, cutting and staining was performed by the Melbourne Histology Platform, The University of Melbourne (Parkville, Australia). Histological descriptions of gonad morphology in salmonids were used to determine phenotypic sex for each sample^40,41^.

### Genomic DNA extraction

Muscle tissue was thawed, blotted dry of excess RNA*later* on a Kimwipe (Kimberly-Clark Professional, USA) and immersed in 1X phosphate-buffered saline solution (Bio-Rad, USA) for 2 hours prior to gDNA extraction. Following, gDNA was extracted using the DNeasy Blood & Tissue Kit (Qiagen, Germany) following the manufacturer’s specifications. All extractions were normalised to 5 ng/µl using a spectrophotometer (NanoDrop, Thermo Fisher Scientifc, USA) and quality checked by visualisation on 1% 1X TAE agarose gel with GelRed (Gene Target Solutions, Australia).

### Conventional PCR-based assay

A multiplex PCR-based test for identifying the genotypic sex of Atlantic salmon was adapted from Eisbrenner, *et al*.^27^. The multiplex consisted of primer pairs for exons 2 and 3 of *sdY* to determine genotypic sex, and primer pairs for *fabp6b* to verify gDNA quality was sufficient for PCR. Primer sequences and amplicon sizes are provided in Supplementary Table S4. Each 25 μl reaction contained 50 ng gDNA, 1.25 units iTaq DNA polymerase (Bio-Rad, USA), 1X iTaq DNA buffer (Bio-Rad, USA), 1.5 mM MgCl_2_ (Bio-Rad, USA), 200 μm dNTP mix (Bio-Rad, USA), 3.45% dimethyl sulfoxide (Merck, Germany), 170 nm exon 2 forward and reverse primers, 1.15 μm exon 3 forward and reverse primers, 400 nm *fabp6b* forward and reverse primers and nuclease-free water (Ambion, USA). All reactions were loaded in triplicate into a hard shell 96 clear-well PCR plate (Bio-Rad, USA), covered with a clear adhesive PCR plate seal (Bio-Rad, USA) and run on a Mastercycler ep gradient S (Eppendorf, Germany). Thermal cycling conditions were as reported by Eisbrenner, *et al*.^27^. Three no template control (NTC) reactions were included in each PCR run. PCR products for each reaction were visualised on a 2% 1X TAE agarose gel with GelRed alongside a 50 bp DNA ladder (Thermo Fisher Scientific, USA). Amplicon presence was determined using Image Lab Software (Bio-Rad, USA) with band detection sensitivity set to 100%. Positive amplification of the target for each sample was given when a band was detected in one or more of the triplicate reactions. Results denoting male and female genotype for this PCR assay are outlined by Eisbrenner, et al^27^. Briefly, male genotype was indicated when products for all 3 primer pairs were amplified. Female genotype was denoted by all other results provided *fabp6b* primers produced an amplicon.

### Real-time qPCR assays

Real-time qPCR assays were developed to determine the quantities of exons 2, 3 and 4 of *sdY* in Atlantic salmon gDNA, and are reported in accordance with the Minimum Information for Publication of Quantitative Real-Time PCR Experiments (MIQE)^42^. An MIQE checklist is provided in Supplementary Table S5^42^. Primer and hydrolysis probes specific to exons 2, 3 and 4 of Atlantic salmon *sdY* were designed using PrimerQuest Tool (Integrated DNA Technologies, USA) according to the gene sequence provided by http://www.ncbi.nlm.nih.gov for *sdY* in Atlantic salmon (GenBank accession number: KT223110). *sdY* gene structure and amplicon localisation for each assay is described in Fig. 3. Primer specificity was confirmed using primer-BLAST (https://www.ncbi.nlm.nih.gov/tools/primer-blast/). Primer and probe sequences, and amplicon sizes are provided in Supplementary Table S6. Each 20 µl reaction contained 10 ng gDNA, 200 nm forward and reverse primer, 200 nm hydrolysis probe, 1X SsoAdvanced universal probes Supermix (Bio-Rad, USA) and nuclease-free water. All reactions were loaded in triplicate into a hard shell 96 clear-well PCR plate, covered with a clear adhesive PCR plate seal and run on a CFX Connect Real-Time PCR Detection System (Bio-Rad, USA). Threshold was manually set to 150 relative fluorescence units, which was within the exponential phase of each run, and Quantification cycle (C_q_) was determined using CFX Manager software 3.1 (Bio-Rad, USA). Positive amplification of the target for each sample was given when amplification was detected in one or more of the triplicate reactions. Thermal cycling conditions were 95 °C for 3 minutes, followed by 40 cycles of 95 °C for 15 seconds and 60 °C for 60 seconds. Three NTC reactions were also included in each real-time qPCR run, and showed no amplification throughout the study except for a single replicate in one exon 4 run (C_q_ 38.76). PCR efficiency for exon 2, 3 and 4 assays were determined by four five-fold serial dilutions of pooled gDNA from two *sdY*-positive samples, and was 94.6%, 94.3% and 92.8% respectively. Target amplicon size and sequence was verified by visualising real-time qPCR products for each assay on 2% 1X TAE agarose gel with GelRed alongside a 50 bp ladder and Sanger sequencing, respectively. However, the amplicon sequence for the exon 3 assay was unable to be identified due to being too small for Sanger sequencing.

### Absolute quantification

A 397 bp gBlocks Gene Fragment (Integrated DNA Technologies, USA) containing the target amplicons for exon 2, 3 and 4 real-time qPCR assays was designed for use as a standard curve in each assay. The dried gBlocks Gene Fragment pellet was re-suspended with nuclease-free water to obtain a concentration of 8.55 ng/μl, as determined by measurement with a spectrophotometer. The total number of copies/μl for each target in this solution was determined as 1.96 ×10^10^ using the following formula: number of copies/μl = (6.02 ×10^23^ x DNA (g/μl))/(DNA length (bp) x 660)^43^. This formula assumes that the average weight of a base pair is 660 Da^44,45^. To determine the linear dynamic range (LDR) for each assay, a ten-point standard curve was constructed by performing 10-fold serial dilutions of the gBlocks Gene Fragment from 1.96 × 10^9^ copies/μl to 1.96 × 10^0^ copies/μl. All standards were run in triplicate following the conditions outlined above, and the LDR was defined between the lowest and highest concentration for which all 3 replicates amplified accurately with less than 1 cycle of deviation^42^. The LDR for exon 2, 3 and 4 assays was 3.93 × 10^9^ to 3.93 × 10^1^ copies/reaction (Supplementary Data S1). For each assay, a six-point standard curve was constructed by performing 10-fold serial dilutions from 1.96 ×10^6^ copies/μl to 1.96 × 10^1^ copies/μl. The standard curve was run six to nine times for each assay in runs containing gDNA samples. For each run, new serial dilutions were performed from a single use aliquot of a 1.96 × 10^9^ copies/μl stock solution.

To address the significant effects minor run-to-run variations in standard preparation and loading can have on number of copies estimates, a master standard curve approach was used^46,47^. For each assay, highly fitted master standard curves were produced by plotting the mean C_q_ of all replicates for each standard (independent of run) against the log-transformed known number of copies/reaction^48,49^ (Supplementary Data S2). The equations of the master standard curves were then used to transform C_q_ values to number of copies/reaction for gDNA samples. Data was normalised to template concentrations as determined by spectrophotometer, and presented as number of copies/10 ng gDNA. PCR efficiencies calculated from the master standard curves (92.1%, 91.0% and 89.3% for exon 2, 3 and 4 assays, respectively) were similar to those determined from gDNA, suggesting they provide accurate number of copy estimates. Intra-assay coefficient of variation as a percentage (c.v %) for exon 2, 3 and 4 assays was 13.92, 6.29 and 11.06, respectively, whilst inter-assay c.v % was 2.86, 4.54 and 7.52.

### Statistical analysis

All statistical analysis was performed through R 3.3.3 (www.r-project.org). Data was checked visually for homogeneity of variance by examining the residuals plotted against the predicted values. Quantile-quantile plots were used to see if the data was normally distributed. A two-tailed Welch’s t-test (variances were not treated as equal) was used to compare the mean number of copies for exon 3 and exon 4 in phenotypic males and phenotypic females (with levels within the LDR). For exon 2, a one-tailed one sample t-test was used to test if the mean for phenotypic males was greater than 55.20 (the number of copies/10 ng gDNA in the single phenotypic female with levels within the LDR). A two-tailed independent t-test (variances were treated as equal) was used to test for differences in the means of phenotypic males grouped according to reproductive developmental stage. Statistical significance was considered at a level of p < 0.05.

## Supplementary information

Supplementary Information.

Supplementary Information2.

Supplementary Information3.
