# Publisher Correction: Discordance for genotypic sex in phenotypic female Atlantic salmon (*Salmo salar*) is related to a reduced sdY copy number

**DOI:** 10.1038/s41598-020-69049-0

**Published:** 2020-07-15

**Authors:** Morgan S. Brown, Brad S. Evans, Luis O. B. Afonso

**Affiliations:** 1 School of Life and Environmental Sciences, Centre for Integrative Ecology, Deakin University Warrnambool Campus, Warrnambool, Victoria 3280 Australia; 2 Breeding & Research, Tassal Operations, Hobart, Tasmania 7000 Australia

Correction to: *Scientific Reports* https://doi.org/10.1038/s41598-020-66406-x, published online 15 June 2020

In this Article, the legend of Figure 2 is incorrect:

“Quantification of *sdY* by real-time qPCR. Number of *sdY* copies detected within the linear dynamic range (LDR) of the real-time qPCR assay in phenotypic males ( ) and phenotypic females ( ), and outside of the LDR in phenotypic females ( ). indicates a phenotypic female with no *sdY* copies detected. (a) data for all 176 Tasmanian Atlantic salmon used in this study; (b) magnified view of individuals with low numbers of target copies.”

Should read:

“Quantification of *sdY* by real-time qPCR. Number of *sdY* copies detected within the linear dynamic range (LDR) of the real-time qPCR assay in phenotypic males ( ) and phenotypic females ( ), and outside of the LDR in phenotypic females ( ). indicates a phenotypic female with no *sdY* copies detected. (a) data for all 176 Tasmanian Atlantic salmon used in this study; (b) magnified view of individuals with low numbers of target copies.”

