# Supplementary material for: Discordance for genotypic sex in phenotypic female Atlantic salmon (Salmo salar) is related to a reduced sdY copy number
Source: Sci Rep. 2020 Jun 15;10:9651. doi: 10.1038/s41598-020-66406-x (PMC7296011; doi:10.1038/s41598-020-66406-x)
Supplement: Supplementary file 2 — Supplementary Information2. [file 41598_2020_66406_MOESM2_ESM.pdf]

Title

Discordance for genotypic sex in phenotypic female Atlantic salmon (*Salmo salar*) is related to a reduced *sdY* copy number

Authors

Morgan S. Brown, Brad S. Evans, Luis O.B. Afonso

Supplementary Table S1. Real-time qPCR results for repeated DNA extractions from phenotypic female samples.

	1st extraction and qPCR run			2nd qPCR extraction and run		
**Sample ID**	**Exon 2**	**Exon 3**	**Exon 4**	**Exon 2**	**Exon 3**	**Exon 4**
7	0	<LDR	<LDR	NT	0	<LDR
11	0	0	<LDR	NT	NT	0
19	<LDR	81.49	57.63	0	0	0
29	0	<LDR	0	NT	<LDR	NT
33	<LDR	0	<LDR	0	NT	0
38	<LDR	<LDR	<LDR	<LDR	70.56	70.55
41	55.20	142.56	121.05	<LDR	<LDR	<LDR
52	<LDR	<LDR	<LDR	0	<LDR	<LDR
54	<LDR	68.83	56.94	0	0	<LDR
56	0	0	<LDR	NT	NT	0
58	0	<LDR	0	NT	0	NT
62	0	<LDR	<LDR	NT	0	0
65	0	<LDR	<LDR	NT	0	0
71	0	<LDR	<LDR	NT	0	<LDR
72	0	0	<LDR	NT	NT	<LDR
88	0	0	<LDR	NT	NT	<LDR
95	0	0	<LDR	NT	NT	<LDR
98	0	<LDR	<LDR	NT	<LDR	<LDR
105	0	<LDR	<LDR	NT	<LDR	<LDR
113	<LDR	54.32	66.05	<LDR	<LDR	<LDR
124	0	<LDR	<LDR	NT	<LDR	<LDR
132	0	0	<LDR	NT	NT	0
135	<LDR	<LDR	<LDR	0	0	<LDR
144	0	0	<LDR	NT	NT	0
147	0	0	<LDR	NT	NT	0
155	<LDR	<LDR	<LDR	0	<LDR	0
156	<LDR	0	<LDR	0	NT	0
163	0	0	<LDR	NT	NT	<LDR
166	0	0	<LDR	NT	NT	<LDR
196	0	0	<LDR	NT	NT	<LDR

Data presented as copies/10ng DNA. <LDR, below the linear dynamic range of the real-time qPCR assay; NT, not tested.

Supplementary Table S2. Real-time qPCR results for repeated DNA extractions from phenotypic male samples.

	1st extraction and qPCR run			2nd qPCR extraction and run		
**Sample ID**	**Exon 2**	**Exon 3**	**Exon 4**	**Exon 2**	**Exon 3**	**Exon 4**
5	1682.77	2193.17	2251.36	2829.33	3866.89	3358.56
6	902.52	1100.50	1028.64	982.64	1513.59	1234.79
8	1346.96	1746.99	1533.00	1419.15	2053.73	1809.66
49	3503.12	4148.83	3794.20	3095.86	3850.65	3608.86
148	2250.83	2745.02	2741.68	2665.97	3559.98	3146.29
149	2000.48	2418.48	2511.30	2529.80	3514.80	2861.36
151	2986.27	4104.24	3745.72	2827.25	3847.43	3551.85
152	2977.35	4070.55	3867.90	2238.09	3413.04	2989.09
153	3158.85	3903.88	3950.63	3175.94	4601.41	4071.22
154	1999.48	2535.29	2531.31	2995.45	4150.47	3895.92

Data presented as copies/10ng DNA.

Supplementary Table S3. Real-time qPCR results for archived fin samples from known sdY-negative phenotypic males.

Sample ID	Exon 2	Exon 3	Exon 4
A1	0	0	0
A2	0	0	0
A3	0	0	0
A4	0	0	0
A5	0	0	0
A6	0	0	0
A7	0	0	0
A8	0	0	0
A9	0	0	0
A10	0	0	<LDR
A11	0	0	0
A12	0	0	0
A13	0	0	<LDR
A14	0	0	0
A15	0	0	0
A16	0	0	<LDR
A17	0	0	0
A18	0	0	0
A19	0	0	0
A20	0	0	0

Data presented as copies/10ng DNA. <LDR, below the linear dynamic range of the real-time qPCR assay.

Supplementary Table S4. Primers for multiplex PCR-based test.

Target	Forward sequence (5' → 3')	Reverse sequence (5' → 3')	Amplicon size
sdY exon 2	TGATGGATGGGATCCCCGTCATCTCTCTCCCAAAG	TAGAGCTTAAAACCACTCCACCCTCCATGAGGGA	123 bp
sdY exon 3	AGTTGGAACGCTTCAGCAGAGCAGATGG	AGATTGGTGCACTGAGTGATGAGTCTTGTCC	93 bp
fabp6b	AATTACGATGAGTTTCTGGAGGCAA	CTTTCCGATGGTGAATTTGTTAGTCAA	457 bp

Supplementary Table S5. MIQE checklist for the reporting of real-time qPCR assays.

**Item to check**	**Importance**	**Reported**
Experimental design		
Definition of experimental and control groups	E	Yes
Number within each group	E	Yes
Assay carried out by the core or investigator's laboratory?	D	No
Acknowledgment of authors' contributions	D	Yes
Sample		
Description	E	Yes
Volume/mass of sample processed	D	No
Microdissection or macrodissection	E	No
Processing procedure	E	Yes
If frozen, how and how quickly?	E	N/A
If fixed, with what and how quickly?	E	Yes
Sample storage conditions and duration (especially for FFPE samples)	E	Yes
Nucleic acid extraction		
Procedure and/or instrumentation	E	Yes
Name of kit and details of any modifications	E	Yes
Source of additional reagents used	D	No
Details of DNase or RNase treatment	E	Yes
Contamination assessment (DNA or RNA)	E	Yes
Nucleic acid quantification	E	Yes
Instrument and method	E	Yes
Purity (A260/A280)	D	No
Yield	D	No
RNA integrity: method/instrument	E	N/A
RIN/RQI or Cq of 3' and 5' transcripts	E	N/A
Electrophoresis traces	D	N/A
Inhibition testing (Cq dilutions, spike, or other)	E	Yes
Reverse transcription		
Complete reaction conditions	E	N/A
Amount of RNA and reaction volume	E	N/A
Priming oligonucleotide (if using GSP) and concentration	E	N/A
Reverse transcriptase and concentration	E	N/A
Temperature and time	E	N/A
Manufacturer of reagents and catalogue numbers	D	N/A
C_qs with and without reverse transcription	D	N/A
Storage conditions of cDNA	D	N/A

**Item to check**	**Importance**	**Reported**
qPCR target information		
Gene symbol	E	Yes
Sequence accession number	E	Yes
Location of amplicon	D	Yes
Amplicon length	E	Yes
In silico specificity screen (BLAST, and so on)	E	Yes
Pseudogenes, retropseudogenes, or other homologs?	D	N/A
Sequence alignment	D	No
Secondary structure analysis of amplicon	D	No
Location of each primer by exon or intron (if applicable)	E	Yes
What splice variants are targeted?	E	No
qPCR oligonucleotides		
Primer sequences	E	Yes
RTPrimerDB identification number	D	N/A
Probe sequences	D	Yes
Location and identity of any modifications	E	N/A
Manufacturer of oligonucleotides	D	Yes
Purification method	D	No
qPCR protocol		
Complete reaction conditions	E	Yes
Reaction volume and amount of cDNA/DNA	E	Yes
Primer, (probe), Mg^{2+}, and dNTP concentrations	E	Yes
Polymerase identity and concentration	E	Yes
Buffer/kit identity and manufacturer	E	N/A
Exact chemical composition of the buffer	D	N/A
Additives (SYBR Green I, DMSO, and so forth)	E	N/A
Manufacturer of plates/tubes and catalog number	D	Yes
Complete thermocycling parameters	E	Yes
Reaction setup (manual/robotic)	D	No
Manufacturer of qPCR instrument	E	Yes
qPCR validation		
Evidence of optimization (from gradients)	D	No
Specificity (gel, sequence, melt, or digest)	E	Yes
For SYBR Green I, C_q of the NTC	E	N/A
Calibration curves with slope and y intercept	E	No
PCR efficiency calculated from slope	E	Yes
CIs for PCR efficiency or SE	D	No
r^2 of calibration curve	E	No
Linear dynamic range	E	Yes

Item to check	Importance	Reported
Cq variation at LOD	E	No
CIs throughout range	D	No
Evidence for LOD	E	No
If multiplex, efficiency and LOD of each assay	E	N/A
Data analysis		
qPCR analysis program (source, version)	E	Yes
Method of C_q determination	E	Yes
Outlier identification and disposition	E	N/A
Results for NTCs	E	Yes
Justification of number and choice of reference genes	E	N/A
Description of normalization method	E	Yes
Number and concordance of biological replicates	D	Yes
Number and stage (reverse transcription or qPCR) of technical replicates	E	Yes
Repeatability (intraassay variation)	E	Yes
Reproducibility (interassay variation, CV)	D	Yes
Power analysis	D	No
Statistical methods for results significance	E	Yes
Software (source, version)	E	Yes
C_q or raw data submission with RDML	D	No

E, essential information; D, desirable information; FFPE, formalin-fixed, paraffin-embedded; RIN, RNA integrity number; RQI, RNA quality indicator; GSP, gene-specific priming; dNTP, deoxynucleoside triphosphate; LOD, limit of detection

Supplementary Table S6. Primers and hydrolysis probes for real-time qPCR assays.

**Target**	**Forward sequence (5' → 3')**	**Reverse sequence (5' → 3')**	**Probe sequence (5' → 3')**	**Amplicon size**
sdY exon 2	AGAGGAGGTGCTTAGTC	AGAGGAGATGGGAATGG	TGATGTCAGAATTGCCTACAAGCC	130 bp
sdY exon 3	CTTCAGCAGAGCAGATG	GTGCACTGAGTGATGAG	TGGGTTCAGCCTATGGTTCGGACA	77 bp
sdY exon 4	CTCTCCTGGAGTCTGAAA	GGAGGAGAGGTGATTAGG	ATGCCACAGCCCTTTCCACCAC	118 bp
